# Nanopipettes as a Potential Diagnostic Tool for Selective Nanopore Detection of Biomolecules

**DOI:** 10.3390/bios14120627

**Published:** 2024-12-19

**Authors:** Regina M. Kuanaeva, Alexander N. Vaneev, Petr V. Gorelkin, Alexander S. Erofeev

**Affiliations:** 1 Research Laboratory of Biophysics, National University of Science and Technology “MISIS”, 119049 Moscow, Russia; 2 Chemistry Department, Lomonosov Moscow State University, 119991 Moscow, Russia

**Keywords:** nanopipette, nanopore, single molecule, molecular diagnostics, solid-state nanopore

## Abstract

Nanopipettes, as a class of solid-state nanopores, have evolved into universal tools in biomedicine for the detection of biomarkers and different biological analytes. Nanopipette-based methods combine high sensitivity, selectivity, single-molecule resolution, and multifunctionality. The features have significantly expanded interest in their applications for the biomolecular detection, imaging, and molecular diagnostics of real samples. Moreover, the ease of manufacturing nanopipettes, coupled with their compatibility with fluorescence and electrochemical methods, makes them ideal for portable point-of-care diagnostic devices. This review summarized the latest progress in nanopipette-based nanopore technology for the detection of biomarkers, DNA, RNA, proteins, and peptides, in particular β-amyloid or α-synuclein, emphasizing the impact of technology on molecular diagnostics. By addressing key challenges in single-molecule detection and expanding applications in diverse biological areas, nanopipettes are poised to play a transformative role in the future of personalized medicine.

## 1. Introduction

Nanopipettes are a subtype of solid-state nanopores that are commonly fabricated from quartz or borosilicate glass capillaries using a laser puller [1,2,3,4]. Micro- and nanopipettes have been used mainly in the fields of cell biology for delivering materials and electrophysiology [5,6,7]. In particular, nanopipettes have become useful tools for such applications due to their precision, inexpensive cost, and multifunctionality. Nowadays nanopipettes are used in imaging, including living cells and various nanomaterials by scanning ion-conductance microscopy (SICM) and scanning electrochemical microscopy (SECM) [8,9,10,11,12].

Nanopipettes and nanopipette-based sensors also play a significant role in cell biology and bionanotechnology by overcoming technical challenges in probing biochemical processes within single living cells and organisms and provide nanometer spatial resolution, millisecond time resolution, high specificity, and sensitivity [1,13,14,15,16,17]. Their unique shape, small size, and excellent electrical properties facilitate obtaining high-resolution topographic images of live cells under physiological conditions [18,19]. These characteristics of nanopipettes can also be used in single-cell analysis, not only on the surface but also for manipulations inside the cell with high precision; for example, using the aspiration principle, it is possible to study desired metabolites inside the cell [20] or to deliver molecules [21,22,23]. The use of nanopipettes as a base for fabricating nanosensors has significantly increased in recent years, particularly for single-cell analysis [24,25]. Nano- and microsensors serve as electrochemical tools to detect and measure reactive oxygen species, metal ions, neurotransmitters, glucose, and ATP in a single living cell [26,27,28,29,30,31,32,33,34,35,36].

The nanopipette-based nanopore detection technology of biomolecules is one of the most promising and dynamically developing field in biochemistry, molecular biology, and medicine, especially in clinical and laboratory analysis [37] and analytical chemistry [38,39,40,41,42]. With the growing need for highly sensitive and specific analysis methods, nanopipette-based nanopore systems offer opportunities for the highly sensitive detection of single molecules and subsequent diagnostics at the molecular level. Nanopore detection is a promising technology to detect several types of proteins, DNA, microRNA simultaneously [37,43,44,45,46,47,48,49,50], or even trace post-translational modifications in the peptide, which is critical for its native function [51]. The use of nanopipettes provides rapid sample preparation, which speeds up the diagnostic process. Due to their small size, nanopipettes can be integrated into portable devices [52].

**Figure 1:**
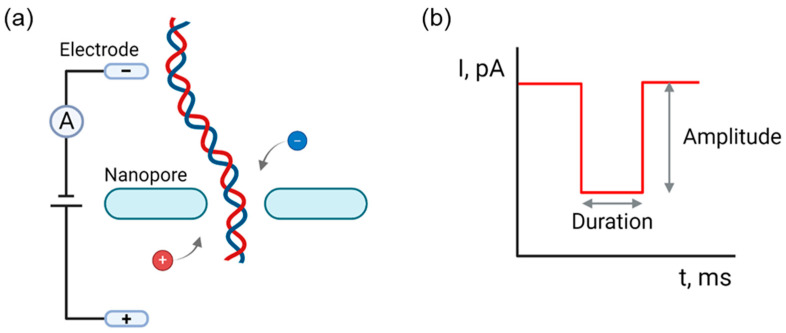
Nanopore detection. (**a**) Experimental principle of nanopore detection. Two baths filled with an electrolyte solution, typically a buffered salt solution, are separated by a single nanopore (or nanopipette). Ag/AgCl electrodes are immersed in the bath and a constant voltage bias is applied across the nanopore. When a molecule translocates through the nanopore, the ionic current typically decreases. (**b**) Characteristics of translocation event. The amplitude and duration (dwell time) of the current reduction during translocation can provide critical insights about biomolecules. Created with Biorender.com.

The principle of nanopore detection by nanopipette relies on resistive pulse sensing (RPS), also known as the Coulter principle. The electric field is generated by applying a voltage bias across two electrodes (typically Ag/AgCl) in two baths separated by a nanopore (Figure 1a). This setup ensures a constant flow of ions in the electrolyte solution. When a charged molecule passes through the nanopore, it temporarily restricts the ion flow, causing a measurable change in the electric current. According to this principle, charged particles or molecules in a salt-buffered solution (e.g., NaCl, KCl, LiCl, or PBS) create electrical disturbances when subjected to an electric field. These disturbances are proportional to the size, charge, and conformation of the particles or molecules [31]. Each event of a molecule translocating through the nanopore is commonly characterized by three key parameters: current amplitude (current blockade), dwell time, and shape of the translocation event. The change frequency of the ion current signals is proportional to the number of particles, the value of the ionic current signal is proportional to the volume of the substance, and its residence time can reflect the length of the molecule. These parameters provide valuable information about the molecule’s characteristics, such as its charge; size; conformation; dipole moments; and, in some cases, even its sequence of the DNA or peptide (Figure 1b) [53,54,55]. For example, DNA sequencing can be achieved by detecting the unique events caused by different nucleotides, allowing us to read the sequence of bases [56]. Similarly, protein sequencing analyzes the current blockades formed by amino acids [57].

Ionic strength and pH affect the properties of a biomolecule’s translocation, the interaction of electrophoretic and electroosmotic flows (EPF and EOF, respectively), and the nanopore surface charge [58]. If the biomolecule is significantly smaller than the nanopore diameter, its translocation may not induce detectable changes in the ionic current. Conversely, if the biomolecule exceeds the pore dimensions, it may obstruct the nanopore entirely, resulting in a complete blockade of ion flow [59].

**Figure 2:**
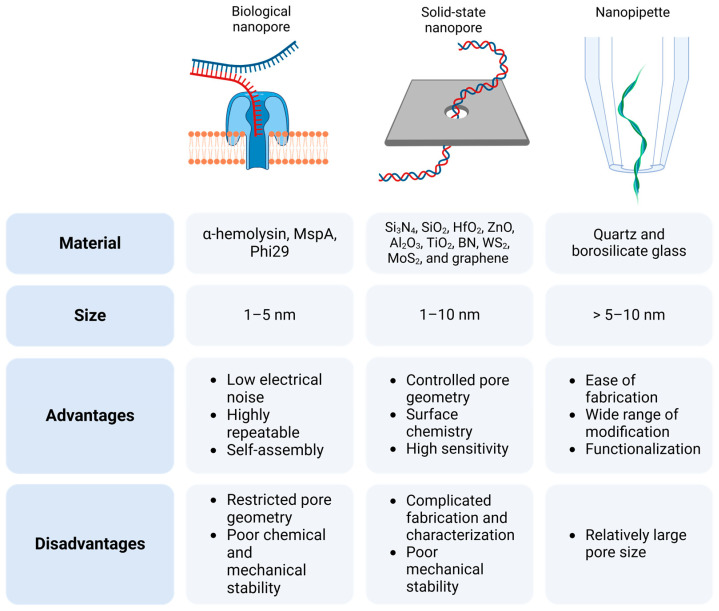
Comparison of biological-, solid-state-, and nanopipette-based nanopores: key materials, advantages, and limitations. Created with Biorender.com.

**Figure 3:**
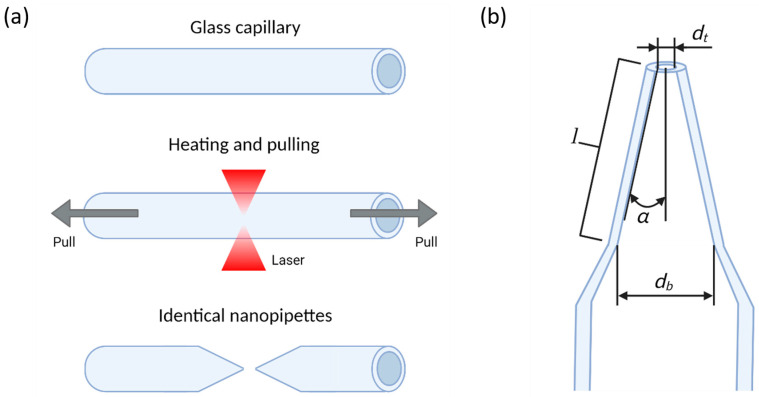
Schematic representation of nanopipette fabrication and characterization. (**a**) Process of pulling a nanopipette. (**b**) Characteristics of the nanopipette, where *l* is the pore length, *d_b_* and *d_t_* are the base and tip diameters of the truncated cone, and α is the half-cone angle. Created with Biorender.com.

A nanopore is a pore (1–100 nm) in a membrane or in a reservoir. In general, nanopores can be divided into two types, biological and synthetic, by the material of the components. A biological nanopore is a pore-forming protein (α-hemolysin, aerolysin, Phi29, MspA) embedded in a lipid bilayer [60]. A solid-state nanopore is a pore in a synthetic membrane from inorganic material, or it can also be a pore in a glass reservoir as a nanopipette (Figure 2). The nanopipette can be distinguished as a third type of nanopore due to its difference from the rest, which is covered below (Figure 3).

Biological nanopores have the smallest possible size among different types of nanopores. Biological nanopores are often used for DNA sequencing or for the characterization of peptide monomers [61,62]. The transmembrane protein α-hemolysin is commonly used as a nanopore, and can independently integrate into the lipid bilayer, forming a pore of 1.4 nm [60,63]. One of the main advantages of biological nanopores is their high reproducibility. Due to their protein nature, each pore has the same structure and size, which ensures a high measurement accuracy. An additional advantage is the ability to modify the protein structure of the pore using genetic engineering methods [64], which allows for creating pores with specified properties, for example, with increased selectivity for certain molecules. Despite these advantages, the main disadvantage is low stability. Protein pores are sensitive to changes in temperature, pH, and other environmental factors, which limit their use. In addition, the size of biological pores is limited, which complicates the analysis of large molecules [65,66].

Solid-state nanopores are artificially created pores (1–10 nm) in thin membranes [40,67]. Unlike biological nanopores, solid-state nanopores are made of various inorganic materials, such as Si_3_N_4_, SiO_2_, HfO_2_, ZnO, TiO_2_, WS_2_, MoS, and track-etched polycarbonate or graphene membranes [68,69,70]. The fabrication of solid-state nanopores is complex and relatively costly [70].

The use of solid-state nanopores solves the problems of biological nanopores since such nanopores are fabricated using advanced technologies from synthetic materials and can be subsequently modified to prevent the adhesion of biomolecules. The advantages of solid-state nanopores include their increased stability, controlled small diameter, and the ability to integrate into portable devices. The stability allows measurements to be made over a wider range of conditions, while the controlled pore size allows for the analysis of molecules of varying sizes. On the other hand, solid-state nanopores also have disadvantages. They can generate noise, their fabrication can be complex, and the pore size can be limited [71].

Among other types of nanopores, nanopipettes are simple to manufacture due to their fabrication from a hollow glass capillary by laser pulling. This method enables precise control over the tip diameter and shape, making nanopipettes universal tools. Moreover, the use of glass as a base material ensures chemical stability and optical transparency, which are beneficial for coupled techniques, such as fluorescence microscopy. However, the main disadvantage compared with other nanopores may be the insufficiently small pore diameter in comparison with solid-state nanopores. Currently, nanopipettes are widely used due to their ease of manufacture, exceptional geometry and chemical strength, possibility of functionalization, and wide range of applications. Their appeal lies in features such as exceptional dielectric properties (low capacitance and minimal electrical noise), and simple fabrication processes that do not require a cleanroom. Both chip-based nanopores and nanopipettes have been successfully incorporated into microfluidic systems [72,73,74].

In this review, we present recent advances from the last 5 years in the use of nanopipette-based nanopore technology in detecting different bioanalytes and for various biomedical applications. Additionally, we highlight the growing role of nanopipettes in single-molecule detection and cellular analysis, underscoring their potential to solve problems in molecular diagnostics and biomedical research.

## 2. Fabrication and Characterization of Nanopipette

A nanopipette is a hollow glass tube (borosilicate or quartz glass) with a pore at one tip. The simplest and most common way to make a nanopipette from a glass tube is to use a laser puller. The puller melts a small section in the center of the tube using a CO_2_ laser beam, and then pulling the tube in opposite directions, the melted part is stretched under the influence of external forces and forms two identical conical nanopipettes (Figure 3a).

The method has the advantages of simple operation, low cost, and accurate adjustment of the aperture. Because the material and thickness of the capillaries are different, the pulling parameters can be varied. The diameter of the pore is controlled by parameters such as the power of the laser beam, the heating area, the amount of delay between the heating and stretching, and the force and speed of the stretching. The minimum pore diameter that can be obtained by laser pulling is sub-10 nm [75], while it is most likely to produce a diameter of 30 nm and above. Due to the properties of the glass, the diameter of the fabricated nanopipette can be shrunk by electron irradiation [76], thus size characterization by electron microscopy can modify the size of the nanopipette. A simple way to reduce the nanopipette diameter during fabrication is to pump air out of the capillary using a vacuum pump [22]. Creating conditions inside the capillary close to a vacuum allows for minimizing heat loss to the internal air and contributes to uniform heating of the pipette area. Coating the inner surface of the nanopipette with a functionalized layer can also lead to the shrinking of the pore size and imparting certain properties to the inner surface [76,77]. Another way to fabricate a nanopipette is obtained by chemical etching [78]. It requires a laser-pulled micropipette with a fused tip, which is then chemically etched in hydrofluoric acid solution.

Borosilicate glass has a lower melting point compared with quartz, making the fabrication of borosilicate nanopipettes more controllable and easier using laser pulling. Additionally, nanopipettes made from borosilicate glass tend to be more symmetrical after the pulling process. However, the minimal pore diameter of a borosilicate nanopipette is limited to 6 nm [79], and more often, they are used as micropipettes. A quartz nanopipette is more durable and can be used for measurements inside the cell [80], and its low electrical noise is useful for electrochemistry research [81,82]. Quartz glass does not fluoresce because it does not contain the metals used in other glasses [83].

The nanopore size and the half-cone (α) angle are key parameters of glass nanopipettes, but traditional methods of their characterization (SEM and TEM) are expensive and require additional preparation. As an alternative, it was proposed to use the electrochemical measurement method for a more efficient characterization of nanopipette tips. Since the nanopipette has the shape of a truncated cone as shown in Figure 3b, the electrical resistance of the pore *R_p_* is

(1) $$R_{p}=\frac{1}{\sigma }\int_{x=0}^{l}\frac{1}{A(x)}dx=\frac{4l}{\pi d_{b}d_{t}\sigma },$$

where *σ* is the electrolyte conductivity, *x* is the coordinate along the centerline, *A(x)* is the cross-section at position *x*, *l* is the pore length, and *d_b_* and *d_t_* are the base and tip diameters of the truncated cone ($d_{b}=d_{t}+2l*tg(\alpha )$, where α is the half-cone angle).

As the ion flow moves from the volume of the nanopipette to the tip, the electric field lines converge, which changes the cross-sections of the ion flux. This contributes to the total pore resistance, and is called the access resistance *R_a_*:

(2) $$R_{a}=\frac{1}{2d\sigma }.$$

Thus, the total resistance of the nanopipette *R_n_* is

(3) $$R_{n}=\frac{1}{\sigma }\left(\frac{4l}{\pi d_{t}d_{b}}+\frac{1}{2d_{t}}+\frac{1}{2d_{b}}\right).$$

This electrochemical approach enables the accurate determination of the actual aperture of a glass nanopipette tip in solution while also allowing for the reuse of the nanopipette after testing. Shigyou et al. compared pore diameter measurements obtained through electrochemical ion current resistance with direct geometrical characterization using TEM. Their findings demonstrated a strong agreement between the methods, highlighting that electrochemical measurements provide reliable estimates of the nanopipette pore diameter [84].

Usually, a nanopipette has one barrel along the entire length, but there are also two- and four-channel capillaries that form multiple pores after fabrication [85,86]. A multi-channel nanopipette allows for capturing and controlling a single molecule with several pores simultaneously [86,87]. That is, detection of the DNA with a two-channel nanopipette can increase its translocation time by 2–3 orders compared with a single channel, which provides enhancement of the temporal resolution. It is also possible to study the DNA through a nanobridge: two pores of one nanopipette are connected by a drop of zeptoliter volume through which the DNA moves [88].

## 3. Single-Molecule Nanopore Detection Using Nanopipette

### 3.1. Nanopore Sensing with Bare Nanopipette

Earlier studies were performed using bare nanopipettes without a specific carrier with binding sites for biomolecules, as confirmed by several studies [89,90,91,92,93,94,95]. The technique with a bare nanopipette has certain limitations, particularly in multicomponent solutions, such as biological fluids (e.g., blood plasma, cerebrospinal fluid, saliva). In these systems, the efficiency of bare nanopipettes is significantly reduced, as the absence of additional selective elements complicates the identification of target analytes with nonspecific signals.

Nonetheless, the simplicity of bare nanopipettes offers several advantages. Specifically, it allows for the optimization of the nanopipette configuration and parameters to address narrowly focused research tasks, such as studying electrostatic interactions, and investigating the mechanisms of molecular transport through nanopores. Therefore, despite their limited applicability in complex systems, this approach remains an important tool for fundamental research and the initial stages of analytical platform development.

One of the most popular biomolecules for translocation through a nanopore is DNA. A huge amount of work over the past 20 years has been done using DNA and other nucleic acids [96,97,98,99,100]. One of the reasons for the popularity of DNA for nanopore research is its relatively simple structure and ability to translocate through nanopores under an electric field due to charge. In addition, DNA is often used as a model molecule for nanopore optimization since it allows for standardized experimental conditions and comparative studies.

Despite the large number of studies in the field of DNA translocation through nanopores, recent studies focused on the EOF during λ-DNA translocation through quartz nanopipettes under low-salt conditions. It was demonstrated that the EOF can be the dominant force driving DNA translocation through nanopores, even under conditions where the electrophoretic force acts in the opposite direction [89].

**Figure 4:**
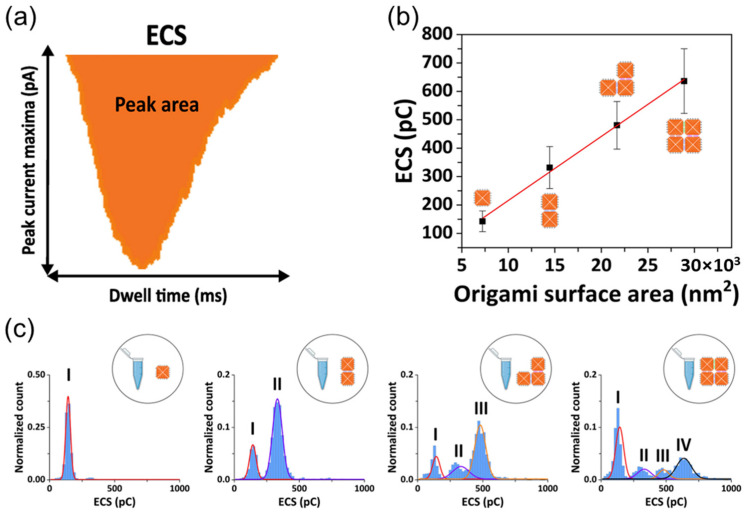
Translocations of DNA origami. (**a**) Schematic representation of the concept of ECS. (**b**) ECS as a function of the DNA origami surface area for the four DNA nanostructure assembled. (**c**) ECS histograms of the DNA origami samples; from left to right: monomer sample, dimer sample, trimer sample, and 2 × 2 sample. Reproduced with permission from [90]. Copyright 2022, Biophysical Society.

Recently, Actis et al. used a nanopipette to analyze and quantify the assembly of supramolecular DNA origami structures. The equivalent charge surplus (ECS) of each translocation event was calculated as the area of the conductive translocation peak (Figure 4a). As expected, the charge increases linearly with the size of the assemblies when identical DNA origami monomers are used (Figure 4b,c). It was demonstrated how nanopipette-based nanopore technology can differentiate different assembly states, ranging from single monomers to more complex structures, such as dimers, trimers, and 2 × 2 structures of DNA [90].

Farajpour et al. used borosilicate nanopipettes to enhance the detection λ-DNA at the liquid–air interface. It was found that reducing the immersed surface area of the pipette and decreasing the nanopore size minimized the capacitive current root mean square (IRMS) noise and increased the signal-to-noise ratio (SNR), while the nanopores with thicker walls demonstrated a better SNR. At the liquid–air interface, the DNA capture frequency increased due to evaporation-induced thermal gradients [91].

For analyzing branched structures of supercoiled plasmid DNA, a special method was developed using quartz nanopipettes. Key findings include the detection of branches by identifying a characteristic current blockade as the branched structures pass through the nanopore, enabling the identification of the branch presence and size. Additionally, the method was applied to study the kinetics of supercoiled DNA linearization catalyzed by the restriction endonuclease NdeI [101].

Freedman et al. developed a highly sensitive protein detection method using quartz nanopipettes and optimized the experimental parameters, including the pore size, electrolyte composition, and applied voltage, to achieve a maximum SNR. Key findings include the development of a “decision tree” for selecting the optimal experimental conditions based on the size and properties of proteins, implementation of high-frequency signal recording with a 100 kHz low-pass filter to minimize distortions, and the development of a method for determining the protein molecular weight by analyzing the relationship between the current blockade, applied voltage, and pore conductance [102].

Nanopipettes can also be used to detect antibodies and antigens; for example, in a recent study, Li et al. focused on the detection of antibodies of glutamate decarboxylase (GADAb), which are an important biomarker for the diagnosis and prognosis of type 1 diabetes. The developed method allowed for identifying the GAD65 antigen, the GADAb antibody, and their complexes based on unique signal characteristics. The ability of the method to differentiate between monoclonal and polyclonal antibodies was confirmed, which provides a new method for the rapid and inexpensive detection of GADAb, which is a preliminary screening indicator for autoimmune type 1 diabetes [94].

**Figure 5:**
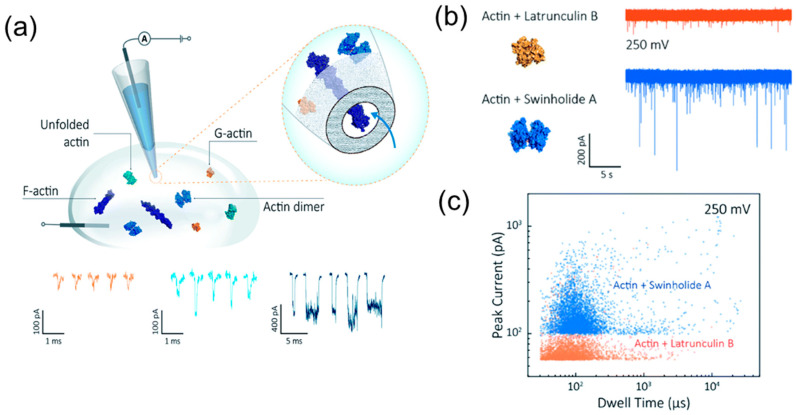
Actin dynamics and its interaction with actin-binding drugs. (**a**) Schematic of experimental setup of protein detection using nanopipette-based nanopore. (**b**) (**Left**) protein models (actin with Latrunculin B bound, and actin with Swinholide A bound). (**Right**) typical current traces for actin bound to different filament inhibitors at 250 mV. (**c**) Scatterplots of current blockades vs. dwell times for both actin monomers and dimers with the same scale at 250 mV. Reproduced from [95] with permission from the Royal Society of Chemistry.

Using bare nanopipettes, Wang et al. demonstrated that single-molecule detection with nanopipette may provide insight into actin dynamics and its interaction with actin-binding drugs [95]. It was shown that each of three forms of actin molecule (monomer, dimer, filament) can be distinguished by its unique translocation signal using an unmodified nanopipette platform (Figure 5a). The interaction of two actin-binding drugs with actin molecules was monitored (Figure 5b,c). While native monomers form oligomers over time, monomers bound with Latrunculin B are locked in its monomeric state and translocate with uniform characteristics. The translocation of dimers bounded by Swinholide A has a deeper signal (current amplitude and translocation time), the values of which increase over time. Thus, the real-time visualization of protein aggregation can be implemented using nanopipettes as a label-free technique for understanding the mechanisms of neurodegenerative diseases.

Long et al. described the use of bare nanopipettes to detect single molecules of glucose oxidase (GOD) [92]. The effect of the nanopipette diameter on the enzyme interaction with its walls and the resulting impact on GOD molecule residence time was studied. In a 67 nm nanopipette, the GOD molecule blocks ion flow during translocation, reducing the current, with a blockade amplitude increasing under a higher voltage, indicating electrophoretic-driven motion. In a 34 nm nanopipette, the signal shifts to a current increase due to electrochemical limitations. In their other study, the same authors focused on developing a strategy for the precise control of single GOD molecule movement inside a nanopipette. A method was suggested to balance the EPF and EOF to increase the retention time of a GOD molecule in the nanopipette region [93].

Also, nanopipettes were used to detect Cas9d10a, a protein with wide applications in genetic engineering and immunology, bound to target DNA strands and unbound Cas9d10a + sgRNA complexes [103].

Table 1 summarizes recent advances in bare nanopipette-based nanopore detection; it highlights studies within the last five years, with different materials and designs for various analyte detections. The cited studies illustrate the capability of nanopipettes in resolving structural details of biomolecules and allow for use in molecular biology and diagnostics. From DNA translocation to protein dynamics and antibody detection, nanopipettes represent a powerful platform for advancing our understanding of molecular mechanisms and developing novel analytical approaches.

### 3.2. Nanopore Sensing with Modified Nanopipette by Recognition Agents

Functionalization of the interior of the nanopipette with recognition agents expanded the utility of nanopore-based detection. Such modification increases the selectivity of nanopipette sensors to enable the discrimination of specific biomolecules, biomarkers, or proteins. By functionalizing the nanopipette walls with aptamers, antibodies, or chemically active compounds, highly specific detection platforms were developed that are capable of determination and quantification of analytes.

Recently, Cao et al. focused on the use of a nanopipette modified with aptamers for analyzing protein–aptamer interactions and detecting carcinoembryonic antigen (CEA) [104]. In this work, the successful modification of nanopores using aptamers was demonstrated and it was showed that the translocation of CEA in the modified nanopores led to a characteristic signal with a large current blockade and long dwell time. The linear range of the method was 2–200 ng/mL. The normal level of CEA in healthy humans is around 3–5 ng/mL. The technique offers benefits such as ease of use, speed, high sensitivity, stability, and cost-effectiveness for identifying CEA, making it highly promising for practical applications in real sample analysis.

**Figure 6:**
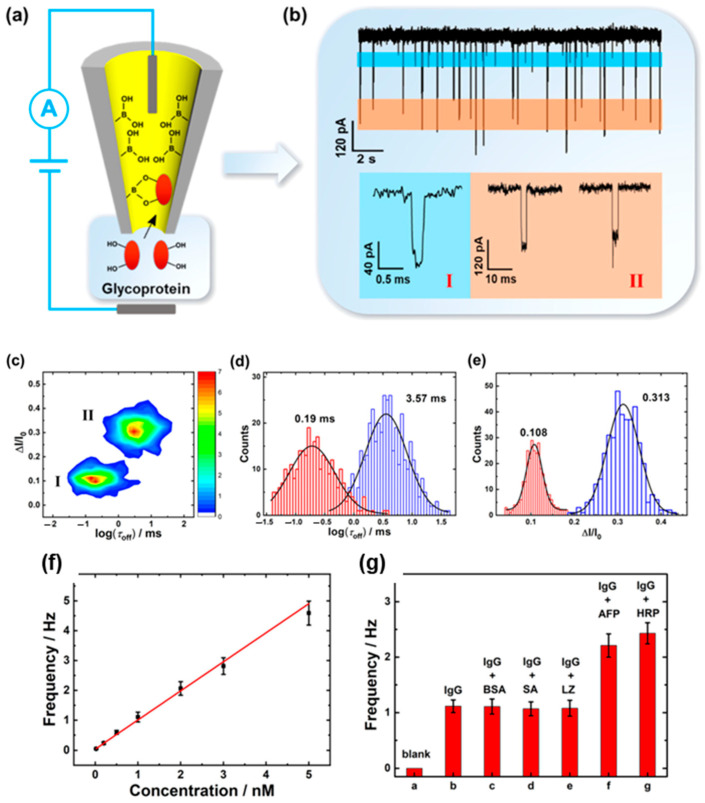
Nanopipette-based nanopore detection of glycoprotein. (**a**) Schematic illustration of the label-free monitoring of single-molecule glycoprotein−boronate affinity. Interaction via a 4-MPBA-modified nanopipette. (**b**) Current−time trace for the presence of 1 nM IgG in 100 mM KCl at +400 mV, (I) translocation of IgG molecules that do not interact with 4-MPBA, (II) translocation of IgG molecules interacted with 4-MPBA. (**c**) Two-dimensional contour plot of ΔI/I_0_ vs. log(dwell time) of the single-IgG current blockade events. (**d**,**e**) Histograms showing the distributions of the logarithmic dwell time (**d**) and ΔI/I_0_ (**e**). Red and blue indicate type I and type II signals, respectively. (**f**) Linear fit plot of the blockade event frequency (type II) and IgG concentrations from 0.02 to 5 nM. (**g**) Blockade event frequency (type II) of the selective detection of IgG in a blank solution, 1 nM IgG, and 1 nM IgG with different coexisting proteins 1 μM BSA, 1 μM SA, 1 μM LZ, 1 nM AFP, and 1 nM HRP. Reproduced with permission from [105]. Copyright 2022, American Chemical Society.

Nanopipettes modified with 4-mercaptophenylboronic acid (4-MPBA) was used for monitoring glycoprotein-borate interactions and detecting immunoglobulin G (IgG) (Figure 6). The approach allows for detecting IgG successfully with high sensitivity and selectivity, showing a linear relationship between the signal frequency and IgG concentration within the range of 0.02−5 nM. The method exhibited high selectivity, as interfering non-glycoproteins (1 µM BSA, streptavidin, and lysozyme) did not affect the detection of IgG, while the glycoproteins (1 nM Alpha-fetoprotein and horseradish peroxidase) could interfere with the detection of IgG to different degrees. Clinical application in real urine samples demonstrated strong agreement with enzyme-linked immunosorbent assay (ELISA) measurements, with recoveries that ranged from 97.2% to 106.6% across multiple concentrations. These results underscore the potential of the approach for sensitive and accurate glycoprotein detection in clinical diagnostics [105].

Chen et al. immobilized Pdx protein onto gold-coated nanopipette for the detection of P450cam monooxygenase. In the nanopore measurements with P450cam in solution, the current blockades were generated by the interactions of Pdx and P450cam, and these interactions prolonged the duration of blockades [106].

A nanopipette-based platform that combines ionic current rectification (ICR) and RPS was developed to achieve the reliable and highly sensitive detection of miRNA-10b, a biomarker for pancreatic cancer [107]. A tetrahedral DNA nanostructure (TDN) within a 30 nm nanopipette was used to enhance the detection sensitivity and linearity, overcoming the limitations of RPS techniques. Zhang et al. achieved the highly sensitive detection of miRNA-10b, with ICR providing a detection limit of 2.4 fM and a linear range of 10 fM to 1 nM. The RPS technique allows for achieving an even lower detection limit of 1 fM and a linear range of 1 fM to 100 pM. The system demonstrated excellent specificity, successfully distinguishing miRNA-10b from closely related miRNAs, such as miRNA-10a and miRNA-21. In biological applications, the method quantified the miRNA-10b expression in single pancreatic cancer (PANC-1) cells and normal (HPNE) cells, with significantly higher levels observed in PANC-1 cells. In plasma samples, miRNA-10b levels in pancreatic cancer patients were approximately 10 times higher than in healthy individuals, with results consistent with the qRT-PCR measurements.

**Figure 7:**
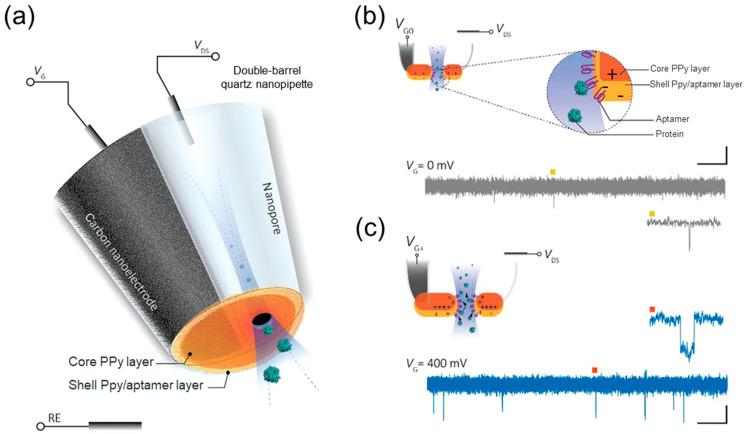
Scheme of an aptamer-functionalized nexFET sensor. (**a**) Double-barrel quartz nanopipette: one barrel is hollow with a nanopore, while the other is filled with pyrolytic carbon. The pipette tip is coated with a thin carbon layer and features a core of PPy and a PPy–aptamer shell. (**b**) In the absence of a gate voltage, the core is positively charged, and the shell is slightly negatively charged, resulting in limited target protein binding. (**c**) Applying a positive gate voltage (V_G_ = 400 mV) enhances the selective thrombin detection, increasing throughput and SNR. Reproduced from [108]. Copyright 2020, the authors. Published by WILEY-VCH.

Ren et al. developed a nanopore extended field-effect transistor (nexFET) sensor functionalized with aptamers for selective protein sensing at the single-molecule level (Figure 7) [108]. The sensor consisted of a double-barrel quartz nanopipette with one bare barrel and the other filled with pyrolytic carbon, forming an integrated electrochemical nanoelectrode. A polypyrrole (PPy) layer is electrochemically polymerized at the tip to serve as the functional sensing surface, and aptamers embedded in the PPy layer provide molecular specificity. By leveraging a gate voltage, the nexFET sensor allows for enhancing the analyte capture rates, SNR, and detection sensitivity, allowing for precise control over the protein translocation through the nanopore. The platform demonstrated high specificity, distinguishing thrombin from other proteins, such as IgG (50 pM).

In summary, nanopipette technology, enhanced with various functional modifications, has shown significant potential for the selective and sensitive detection of biomolecules. Advances in nanopipette designs, such as aptamer-functionalized nanopores, have enabled the precise recognition and quantification of targets like CEA, IgG, P450cam, miRNA-10b, and thrombin. These approaches offer exceptional sensitivity, with detection limits reaching femtomolar levels, and high specificity, as seen in their ability to differentiate between closely related molecules.

The wide versatility of nanopipette-based platforms is underscored by their adaptability for targets from cancer biomarkers to glycoproteins and enzyme interactions. Key advantages include ease of use, rapid detection, high reproducibility, and cost. However, challenges remain, including the complexity of fabrication, the dependency on precise functionalization for each target, and the need for further validation in complex biological fluids. Despite these challenges, nanopipette-based platforms hold immense promise for advancing molecular diagnostics.

### 3.3. Nanopipette-Based Nanopore Sensing by Molecular Carrier with Selective Agents

The specific detection of biomolecules is an acute problem of modern biosensing and diagnostic technologies. It relies on the use of selective agents with high specificity and affinity for the target molecule. These agents, such as antibodies for antigens, streptavidin for biotin, aptamers, and proteins with DNA-binding domains, act as molecular recognition elements that enable the precise identification and quantification of analytes in complex biological fluids. Integrating these agents into detection platforms, researchers can achieve unparalleled selectivity, allowing for the analysis of biomolecules at trace concentrations and even in the presence of structurally similar compounds. This principle forms the basis for a wide range of applications, including disease diagnostics, environmental monitoring, and molecular biology research. In this section, we review recent advances in the development and application of selective agents for biomolecule detection, with a focus on their integration into nanopipette and nanopore-based sensing technologies. We explore their advantages and limitations and discuss their potential to address emerging challenges in molecular detection and diagnostics.

**Figure 8:**
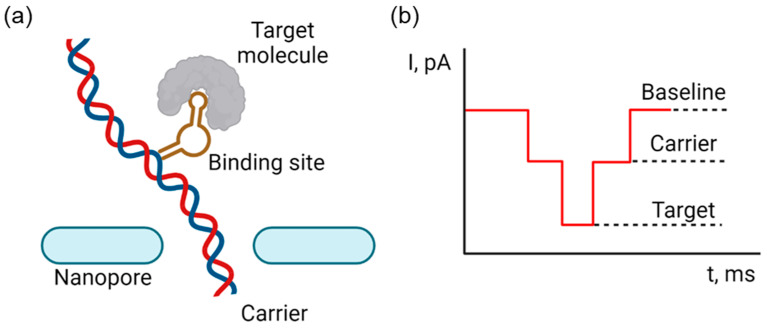
Nanopore detection by a specific carrier. (**a**) Experimental principle of selective detection using molecular agent and a carrier; (**b**) example signal of selective detection. Created with Biorender.com.

Selective single-molecule nanopore detection is provided by a molecular carrier (MC) that binds a biomolecule with high affinity, such as antibody to antigen, aptamer to its recognition sequence, or protein with a DNA-binding domain. The binding site is grafted on a carrier, as shown on Figure 8; it can be a DNA, a nanoparticle (NP), or a fluorescent label, where such a multicomponent system amplifies the signal of translocation and distinguishes it from non-target translocations.

The most advanced binding site for selective detection is the aptamer, an oligonucleotide that can be constructed by combinatorial technique SELEX (systematic evolution of ligands by exponential enrichment) to bind both the carrier and the target molecule [109]. The MC, such as λ-DNA, has a recognizable signal of translocation and a negatively charged backbone, which is strong enough to prevail in molecular transport, and can also have two complementary ends for hybridization of aptamers. Sze et al. [43] used a versatile yet simple concept, such as nucleic acid aptamers grafted onto a DNA carrier, to selectively detect multiple types of protein simultaneously: α-thrombin and acetylcholinesterase (AChE). A thrombin-binding aptamer was used for α-thrombin (37.5 kDa) detection at 1.6 nM, as well as to detect both AChE (280 kDa, using AChE-selective aptamer with a dissociation constant (Kd) of 14 ± 1 pM) and thrombin using a single carrier. The effectiveness of this technique was demonstrated by the successful detection of thrombin in diluted human serum (1:20) using a carrier with three aptamers.

**Figure 9:**
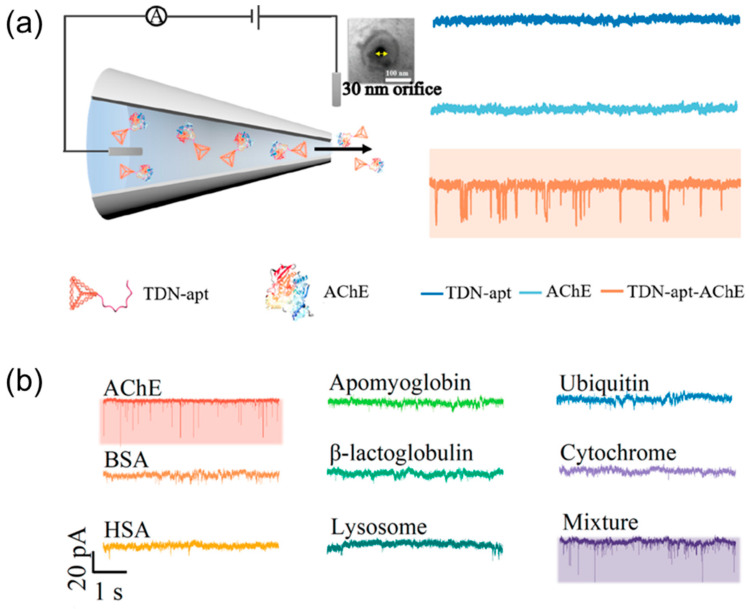
Nanopore detection of AChE using TDN. (**a**) Schematic illustration for the translocation of bare TDN-apt, AChE, and TDN-apt-AChE complex through the nanopipette and the corresponding signals. (**b**) Current−time traces of the translocation of 150 fM TDN-apt interacting with 100 fM AChE and 100 fM of other interferent proteins, as well as their mixture. Reprinted with permission from [110]. Copyright 2022, American Chemical Society.

The carrier itself should not only have detectable geometry but also durability so that it should not change under the influence of EPF and EOF and its translocation signal will be recognizable. To overcome these features of nanopore detection, Zhang et al. [110] used TDN as a carrier (Figure 9). TDN with a grafted AChE-specific aptamer successfully detected target molecules in femtomolar concentrations from both AChE-only and interfering protein buffers. Since AChE is a representative biomarker for Alzheimer’s disease (AD), the TDN-aptamer complex has shown its potential as a sensing platform in real samples, namely, AD patients’ sera, and the detection results were confirmed by ELISA analysis.

In another work, similar constructions of TDN were used as signal amplification probes [111]. Fully assembled TDNs showed the most significant improvements in the SNR and stability during the nanopipette detection, which enabled the precise identification of small biomolecules, such as ATP. Their dwell time increased significantly upon ATP binding, which enhanced the ionic current signals for detection. This demonstrated the ability of TDNs to act as a reliable platform for amplifying signals in nanopore-based assays.

**Figure 10:**
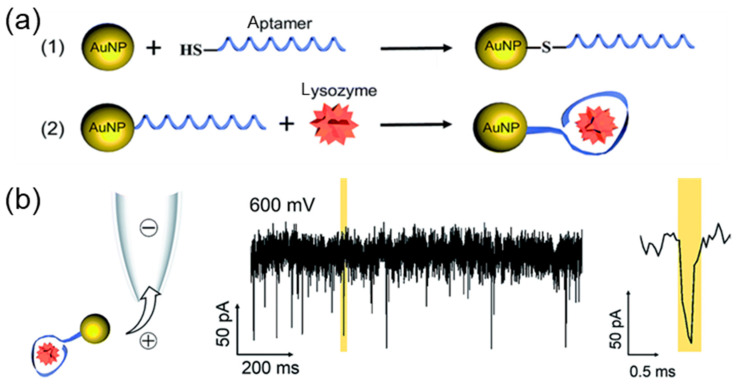
Nanopore detection of lysozyme by AuNPs. (**a**) Structure of AuNPs functionalized with an LBA (1) for detection of lysozyme (2). (**b**) Current–time traces of the AuNP-LBA-lysozyme complex at 600 mV with 100 mM KCl. Reproduced from [112]. Copyright 2017, Royal Society of Chemistry.

The use of DNA as a carrier imposes certain challenges, such as limited signal generation and complex assay conditions. Gold NPs (AuNPs) are simpler and cheaper, are able to bind with thiol via the oxidation–reduction reaction, can be mono- or dimeric, and do not need special conditions for functioning. Lin et al. [112] attached thiol-modified lysozyme-binding aptamer (LBA) to the 5 nm AuNPs carrier using lysozyme as a target protein (Figure 10). Due to the increased excluded volume and reduced charge interference of gold NPs, this molecular system provides improved detection metrics, which have also been validated by using a small-sized protein, such as lysozyme. Such a complex was capable of binding free lysozyme in the range of 125–250 nM from an interfering protein buffer.

**Figure 11:**
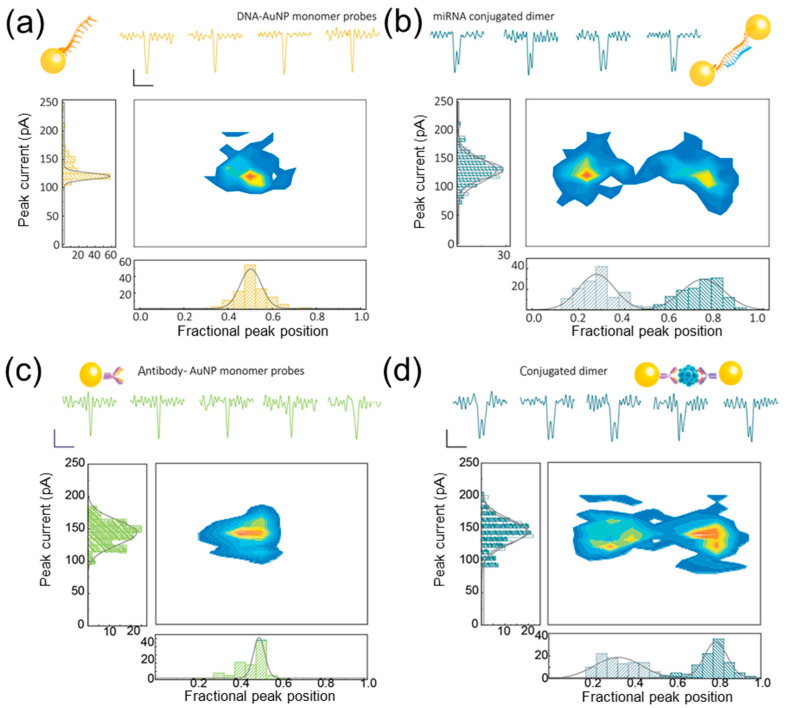
Detection of miRNA-141 and PCT by AuNPs. (**a**) Schematic representation of AuNP monomer miR-141-3p molecular probes with representative individual events (scale bar: vertical 50 pA, horizontal 20 µs), along with the associated statistics. (**b**) Conjugated dimers with miRNA-141 linked between 2 NP monomers. (**c**) AuNP monomeric antibody molecular probes with individual translocation events (scale bar: vertical 50 pA, horizontal 20 µs), along with associated statistics. (**d**) Conjugated antibody dimers with PCT (an antigen). Reproduced from [113]. Copyright 2021, John Wiley and Sons.

To improve the translocation signal of the AuNP, it is possible to use a dimeric system that self-assembles upon the binding of the analyte and produces a doublet in the translocational signal. Ren et al. [113] used AuNP dimers for the detection of microRNA associated with active prostate cancer (miR-141-3p) and procalcitonin, a biomarker of sepsis. Thus, with the addition of target miRNAs (at pM–nM concentration), AuNP dimerization occurs (Figure 11b), resulting in doublet signals, which can be used to estimate the target’s concentration. To detect procalcitonin (14.5 kDa), two groups of AuNP monomers was labelled with two types of antibodies (Figure 11c) so that upon binding to the procalcitonin at the concentration from 0 to 10 ng/mL, the monomeric system self-assembled by forming a “sandwich” (Figure 11d). Thus, the presence (or absence) and concentration of target molecules could be calculated. The method is adaptable for the detection of alternative target molecules by NPs functionalization with desired binding sites.

To improve the selectivity of the detection with an MC, additional physical methods in the form of magnetic separation or fluorescent labels are increasingly used. For example, magnetic NPs (MNPs) with thiolated aptamers for CEA were used by Tang et al. [114] for CEA detection, providing the probe with an aptamer-binding ability by a gold shell, and achieving additional magnetic separation in a complex sample. After the incubation with the CEA (≤1 nM), the magnetic separation of three types of probes occurred: MNP, Apt-MNP, and CEA-Apt-MNP. The selectivity of the CEA–aptamer was verified by detection in interfering protein buffers (blank solution, 1 nM CEA only, and 1 nM CEA with a 1 nM of coexisting proteins). Despite the low detection limit of this work (0.6 ng/mL), which was determined by the binding agent, the proposed technique is distinguished by a multi-stage separation, which is a superior characteristic when working with serums 

**Figure 12:**
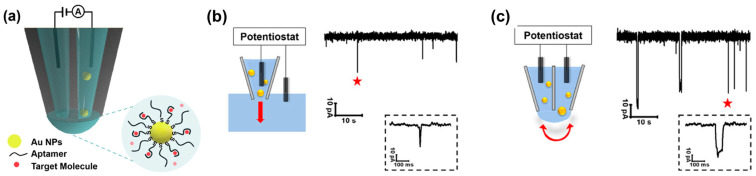
Double-barrel nanopipette-based system for the detection of dopamine, serotonin, and K^+^. (**a**) Schematic illustration of the measurement principle based on a dual nanopipette. Ionic current recordings of the translocations of 20 nm Au-PEG NPs in 100 mM KCl at 400 mV performed in a single nanopipette (**b**) and a dual nanopipette (**c**) (stars highlight representative events in dashed boxes). Reprinted with permission from [115]. Copyright 2022, American Chemical Society.

A double-nanopipette system for the detection of single NPs and small molecules was developed [115]. AuNPs functionalized with aptamers demonstrated the system’s ability to detect dopamine (detection limit: 12.5 nM), serotonin (100 pM), and potassium ions (900 nM) (Figure 12). The enhanced resolution of the dual nanopipette also allowed for the differentiation of NP sizes, with 10 nm and 20 nm AuNPs showing distinct current blockage profiles. The double-nanopipette design creates a confined translocation path that slows down the movement of analytes, enabling more precise measurements of current blockages. Key advantages of the system include its ability to detect both electroactive and non-electroactive molecules without relying on redox reactions, its high sensitivity due to reduced translocation speeds, and its versatility for biosensing applications. However, limitations include the reliance on precise nanopipette fabrication and the requirement for aptamer functionalization, which may demand optimization for each target molecule.

**Figure 13:**
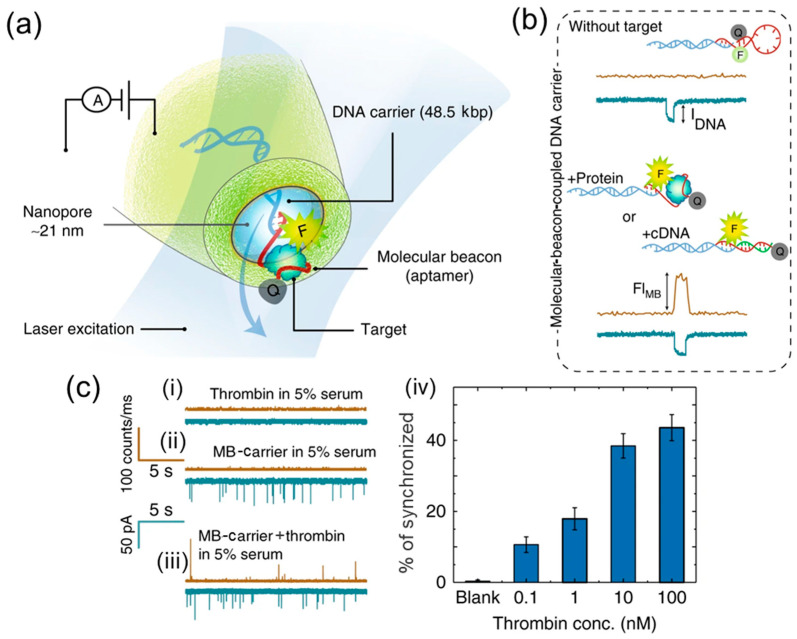
Nanopore electro-optical approach to detect thrombin. (**a**) Schematic of the electro-optical configuration, where a nanopore is integrated with a single-molecule fluorescence confocal microscope. (**b**) MBs are hybridized to a DNA carrier for the single-molecule detection of small oligonucleotides or proteins. Top: without target binding, the signal is only observed in the electrical detection channel. Bottom: when bound to a complementary DNA or protein, a synchronized signal is observed in both channels due to the opening of the MB and the increased distance between the fluorophore and the quencher probes. (**c**) Photon and current time traces are shown for the translocation of (i) thrombin in 5% serum, (ii) MB–carrier in 5% serum, and (iii) MB–carrier bound to thrombin in 5% serum. (iv) Percent synchronization between the optical and electrical channels for thrombin bound to the MB–carrier. Reprinted from [116]. Copyright 2019, the authors.

The use of fluorescent labels in nanopore detection stands out as a groundbreaking approach, combining electrical and optical signals to deliver highly reliable results. This innovative technology leverages molecular beacons (MBs) that use a fluorophore and a quencher to create electrical and optical signals. Cai et al. [116] demonstrated the efficiency of such an electro-optical binding technique for selective single-molecule detection (Figure 13) by the verification of the synchronization of electrical and optical detection, fluorophore sensitivity, sensitivity of the aptamer, and efficiency of this technique for protein detection. The unique design of the MB allows it to be in one of two states: unbound (or inactive, produces only a translocation signal, as shown on Figure 13b, top) and bound (active, synchronization of an optical and translocation signal, as shown on Figure 13b, bottom). Thus, the coincidence of the optical signal with the translocation event signifies that the target biomolecule with activated MB passed through the pore. An MB-modified carrier (10 pM) was used for thrombin detection in experimental (30 nM in 0.1 M KCl) and biological sample (0.1 to 100 nM in urine and serum, as shown on Figure 13c). A synchronized nanopore platform minimizes the false positive detection results that non-combined techniques are prone to.

Based on a similar principle, an innovative platform for the direct detection of miRNAs in serum without requiring RNA amplification or extraction was developed [37]. The platform’s functionality relies on nanopipettes containing molecular probes—size-coded DNA carriers and MBs that fluoresce upon binding to target miRNAs. Each DNA carrier produces a unique current signal as it translocates through the nanopore, enabling the identification of different miRNAs, while the fluorescence signal confirms successful binding.

This method achieves exceptional sensitivity that is capable of detecting miRNAs at femtomolar concentrations, outperforming traditional techniques, like confocal microscopy and electrochemical methods. This level of sensitivity is particularly important for analyzing biological fluids, where the miRNA concentrations are typically very low. The platform also supports multiplexed detection, as demonstrated by the simultaneous identification of miR-141-3p, miR-375-3p, and let-7a. The multiplexing capacity can be further expanded by using longer DNA carriers and a broader range of fluorophores. The platform accurately distinguished patients with active prostate cancer from those in remission by analyzing miR-141-3p and miR-375-3p. The results demonstrated superior sensitivity and accuracy compared with RT-qPCR.

**Figure 14:**
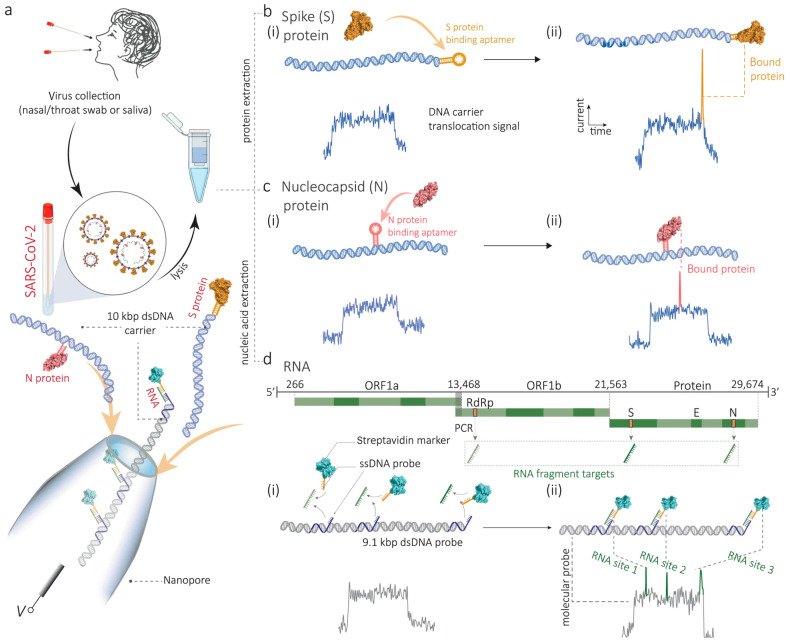
Multiplexed detection of SARS-CoV-2 viral proteins and RNA fragments. (**a**) Schematic of multiplexed detection of SARS-CoV-2 viral proteins and RNA fragments. (**b**) Ion current–time traces for 10 kbp dsDNA probes encoded with an S protein aptamer without bound S protein (i), and when the S protein is bound, a secondary peak occurs at the end (ii). (**c**) Similarly, 10 kbp dsDNA probes encoded with an N protein aptamer (without bound with N protein (i)), display a secondary peak in the middle upon N protein binding (ii). (**d**) A 9.1 kbp DNA probe encoded with sequences complementary to the ORF1b, S, and N genes (i) enables RNA fragment detection, with specific secondary peaks corresponding to each gene (ii). Reprinted from [41]. Copyright 2019, the authors.

Recently, a multiplexed nanopore-sensing strategy for detecting SARS-CoV-2 proteins and RNA fragments was developed [41]. Using position-encoded DNA molecular probes, Ren et al. detected spike (S) and nucleocapsid (N) proteins directly in unprocessed human saliva and also identified RNA fragments from nasal/throat swabs, distinguishing viral variants, such as Alpha, Delta, and Omicron (Figure 14). The limit of detection of 0.2 pM for proteins and 0.01 RNA copies/µL was achieved, outperforming traditional RT-qPCR in sensitivity by two orders of magnitude. Key advantages include the ability to multiplex, simultaneously analyzing different proteins and RNA fragments, and discriminate single-nucleotide polymorphisms, enabling variant identification. This reduces false positives/negatives and improves the accuracy of diagnostic evaluations. Furthermore, the method does not require nucleic acid sequencing, offering rapid and cost-effective pathogen detection in clinical conditions.

In another study, a nanopore-based single-molecule detection platform was developed for accurately identifying the A29 protein of the mpox virus (MPXV) directly in human biofluids [46]. A custom DNA molecular probe integrates an aptamer and an antibody, forming a sandwich structure for enhanced specificity and sensitivity. An 11 fM limit of detection was achieved. The method is capable of distinguishing the MPXV A29 protein from closely related viral proteins, such as Vaccinia A27 and Varicella Zoster proteins, despite their high sequence homology. This innovative method bypasses the need for sample preprocessing, making it a potential tool for point-of-care diagnostics.

**Figure 15:**
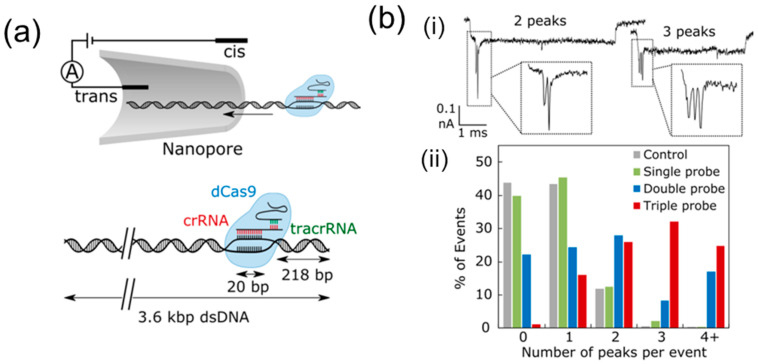
Mapping of recognition sites on DNA. (**a**) Single dCas9 probes bound to 3.6 kbp DNA translocating through a nanopore. (**b**) Double and triple dCas9 probe barcodes on full-length λ-DNA. (i) Example events with two and three peaks due to binding of the double-probe barcode and triple-probe barcode, respectively. (ii) Raw data comparing the number of peaks counted per event after the addition of a single probe, double probe, and triple probe. Reprinted with permission from [117]. Copyright 2019, American Chemical Society.

The mapping of recognition sites on DNA can also be achieved by proteins with a DNA-binding domain, as was accomplished by Weckman et al. [117] using a modified version of the CRISPR-Cas9 system. Since linearized DNA chains can form knots and folds during translocation, it is necessary to create a unique barcode for each target DNA using controllable probes. An inactive Cas9 protein, dCas9, can be designed as a controlled highly specific binding site to map several sequences on one DNA chain or to distinguish between two DNA targets in a mixed pool (Figure 15).

Sandler et al. extended the methodology introduced in [117] by focusing on several other aspects [118]. The newer study systematically evaluated the mismatch tolerance of dCas9 and quantified its ability to discriminate single-nucleotide changes. Sandler et al. incorporated the quantification of relative DNA concentrations in mixtures, an enhancement over Weckman et al.’s purely qualitative identification of DNA barcodes. This advancement allows for more precise and scalable applications in clinical diagnostics. Sandler et al. explored the potential for higher barcode density using smaller nanopores and advanced DNA nanostructures, aiming to increase multiplexing capabilities beyond the simpler double and triple barcodes used in [117]. A significant contribution of the newer work is the development of a screening method for assessing the efficiency and specificity of dCas9 probes. This approach supports tailored probe design for diagnostics and highlights the critical variability in guide RNA sequences.

The supercharged unstructured polypeptides (SUPs) were used as MCs to improve protein detection [44]. By genetically fusing proteins of interest with SUPs, the researchers achieved significant control over the translocation of proteins through nanopores. Cationic SUPs slow down translocation times by up to 44.7-fold, improving the detection accuracy and enabling a detailed analysis of subpeaks in the ionic current. Using quartz nanopipettes with a pore diameter of approximately 12 nm, the system successfully resolved proteins of different sizes and shapes, including eGFP and fluorescent proteins, with distinct subpeak characteristics. Quantitative results include the SUP-enhanced capture rate of proteins, which increased by up to 136-fold compared with the uncharged variants, and Kd = 7.85 nM for the antigen–antibody interactions. A limit of detection of 74 pM was achieved. The advantages of this approach include its tunable genetic design, enhanced temporal resolution, and compatibility with diverse proteins, making it a promising tool for proteomics and diagnostics. However, the method’s reliance on precise SUP fabrication and potential interference from carrier folding highlight areas for further optimization.

Nanopipette-based nanopore sensing by MCs with selective elements represents a significant advancement in biomolecule detection. The approach combines high specificity and sensitivity, enabling the precise analysis of target molecules, even in complex biological fluids. Using carriers such as aptamers, DNA nanostructures, AuNPs, and proteins, researchers achieved enhanced signal resolution, improved detection limits, and the ability to multiplex assays. These innovations hold great promise for applications in diagnostics, offering powerful tools for solving current challenges in single-molecule detection (Table 2).

## 4. Nanopipette-Based Nanopore Technology as a Tool for Studying Protein Aggregation and Misfolding

Due to the complex processes of protein folding into its native state, the disruption in conformational changes leads to the formation of a misfolded protein that does not perform its intended function. In such pathways, an alpha helix native conformational state transitions into a beta sheet, which is a characteristic of an β-amyloid, and the misfolded protein becomes toxic. This transition exposes hydrophobic amino acid residues, which promotes protein aggregation. Some proteins specifically aggregate and have toxic effects in the central nervous system, even though their expression occurs throughout the body [120]. These neurodegenerative diseases include disorders in which abnormal proteins can accumulate intranuclearly, such as polyglutamine in Huntington’s disease [121] and spinocerebellar ataxia [122]; disorders characterized by cytoplasmic inclusions, such as α-synuclein in Parkinson’s disease [123]; disorders in which abnormal proteins accumulate extracellularly in prion diseases [124]; or both intracellularly and extracellularly, such as tau protein and β-amyloid peptide in AD [125,126]. The complex nature of protein misfolding and aggregation are complicated for proper research; it needs a versatile and adaptive analyzing tool. In this case, nanopore detection allows for studying peptides in any stage of aggregation and for continuous measurement at the same time because of the nanopipette’s form, pore’s geometry adjustability, and functionalization by coating, which prevent the fouling and adsorption of highly unstable oligomers.

**Figure 16:**
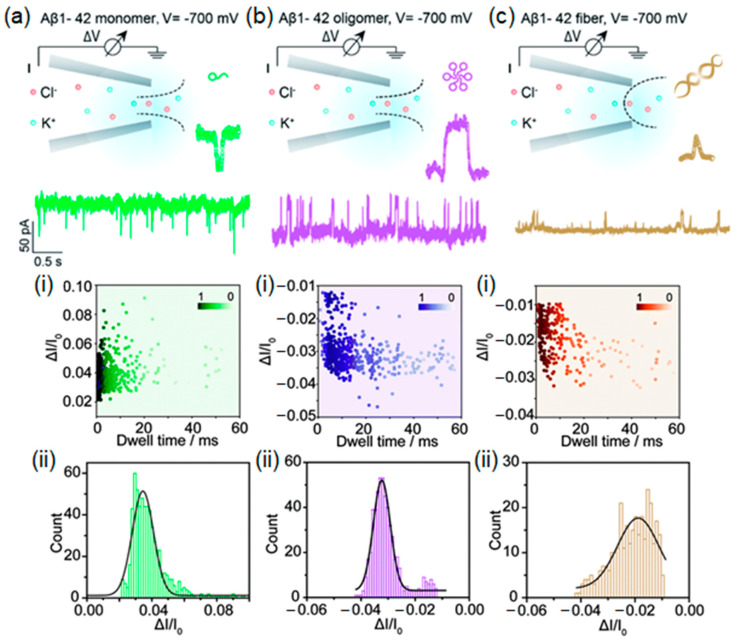
Single-molecule monitoring of Aβ_1–42_ monomers. (**a**) Schematic representation of the single-molecule monitoring of Aβ_1–42_ monomers: (i) event scatter plots and (ii) histograms of the current amplitude versus dwell time for the translocation of Aβ_1–42_ monomers. (**b**) Single-molecule monitoring of Aβ_1–42_ oligomers: (i) event scatter plots and (ii) histograms of the current amplitude versus dwell time for the translocation of Aβ_1–42_ oligomers. (**c**) Single-molecule monitoring of Aβ_1–42_ fibers: (i) event scatter plots and (ii) histograms of the current amplitude versus dwell time for the translocation of Aβ_1–42_ fibers. Reproduced from [127] with permission from the Royal Society of Chemistry.

The single-molecule detection of different types of β-amyloid aggregates can be achieved using a nanopipette through an RPS. Sensing monomers, oligomers, and fibrils in an aqueous solution provides not only an aggregation study as a process but also collects data for early diagnosis nanopipette-based method development. For example, Yu et al. [127] showed that the translocation of a monomer is a spike-like signal, and for an oligomer, it is mostly staircase-like because of its complex form. The translocation of fibril can have a spike-like signal with a shorter duration than monomers, especially in the experiments where the aggregates are placed outside of the nanopipette. It is associated with a discrepancy in the aggregate’s size and pore’s diameter and has the nature of collision or bumping events [128]. One of the most significant findings in this work [127] is an observed loose packing structure of the oligomers, which easily adsorbs onto solid surfaces, and provides direct single-molecule evidence supporting the toxic Aβ oligomer hypothesis. Moreover, the study showed that monomers induce current enhancement due to ion accumulation, while oligomers and fibers mainly cause current blockages due to volume exclusion (Figure 16).

Investigation into the characterization of Aβ fibrils and oligomers using nanopore technology offers a promising approach for the label-free, real-time analysis of β-amyloid structures in solution. As observed by Abrao-Nemeir et al. [129] for fibrils, the results do not correlate significantly with the fibril length and indicate that other factors, such as the fibril width or nanopore dynamics, may play a more substantial role in determining the current signals. The shorter fibrils, sonicated at a higher power, result in a broader distribution of current blockages, aligning with their observed larger width in TEM analysis.

A recent study focused on understanding the secondary nucleation mechanism of β-amyloid peptide aggregation, a key process in AD pathology. By integrating nanopipette-based detection, confocal fluorescence spectroscopy, molecular dynamics simulations, and Thioflavin T fluorescence assays, the role of preformed aggregates (seeds) in driving β-amyloid (Aβ_42_) peptide aggregation was investigated [130].

It was demonstrated that the fragmented seeds were more efficient in accelerating the aggregation process compared with the unfragmented seeds, reducing the half-time of aggregation by 32% to 42% depending on the seed maturity. Nanopipette-based nanopore measurements revealed a wide distribution of oligomer sizes ranging from a few nanometers to 50 nm, with the fragmented seeds promoting the formation of smaller oligomers earlier in the process.

The study’s key advantages include its multi-technique approach, where it provided detailed insights into aggregation kinetics and structural heterogeneity. However, limitations include the complexity of distinguishing on-pathway and off-pathway oligomers and potential challenges in extrapolating in vitro findings to in vivo conditions.

**Figure 17:**
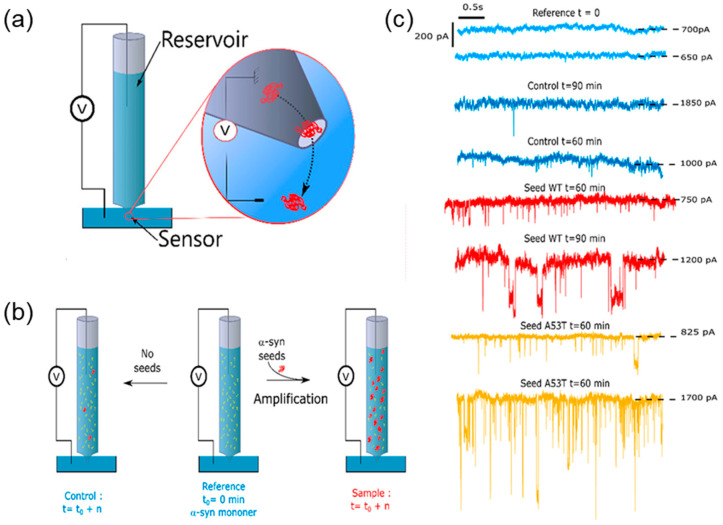
Concept of real-time fast amyloid seeding and translocation (RT-FAST). (**a**) Scheme showing the two parts of a nanopipette: the reservoir where the αS seeds are amplified and the sensor where αS seeds are detected. (**b**) Illustration of the RT-FAST experiments. (**c**) Example of a current trace record extracted from different nanopipettes for reference (light blue), the control not seeded (blue), and the sample seeded with αS WT (red) and A53T mutant (yellow). Reproduced from [132]. Copyright 2022, the authors.

The initial lag phase of peptide aggregation is thermodynamically unfavorable, but the formation of oligomers accelerates the process of aggregation. In this case, each polymer acts as a nucleation point and grows by free monomer incorporation or/and by splitting to several nucleation points [131]. A nanopipette provides an impressive surface-to-volume ratio to enhance the β-amyloid seeding reactions, thus making the nanopipette suitable for use as a platform for simultaneous amyloid seed amplification and detection, which can help to understand kinetic phases of amyloidogenesis. The concept of real-time fast amyloid seeding and translocation (RT-FAST) was implemented by Meyer et al. for both α-synuclein [132] and β-amyloid [133] aggregation studies (Figure 17). In the absence of seeds, initial experiments showed low capture rates of α-synuclein that increased with the rise in the temperature of the experiment, confirming the oligomeric nature of these translocations. This temperature dependency effectively demonstrates the potential of RT-FAST to accelerate the study of protein-misfolding processes. Upon the addition of the seeds and subsequent incubation with α-synuclein, the capture rate increased, and the current distribution broadened, indicating the formation of heterogeneous aggregates. Another advantage of RT-FAST is the reduction in the amount of monomer added and the reaction time (35 μL, 0.1 μM, and 1 h) compared with similar methods.

The RT-FAST assay overcomes the challenges related to the complexity of amyloid-β aggregation pathways [133]. The experiment showed a clear distinction between the control (Aβ monomers alone) and the experimental (Aβ monomers with preformed seeds) conditions. One of the standout features of the RT-FAST assay is its ultrasensitivity, allowing for the detection of amyloid aggregates, even in low concentrations. The ability to monitor the changes in real time provides a powerful tool for researchers investigating the mechanisms of neurodegenerative diseases.

Recent advances include investigating the impact of seed structure on α-synuclein aggregation using nanopipettes, as shown in studies by Charles-Achille et al. [134], where distinct populations of α-synuclein oligomers were observed during the early aggregation phases. Nanopipettes enabled mapping oligomer sizes between 5 and 30 nm and highlighted the seed morphology’s critical role in forming heterogeneous oligomer species. Liu et al. [45] further demonstrated the application of nanopore-based detection to differentiate α-synuclein oligomers directly in the cerebrospinal fluid of Parkinson’s disease patients. Their study achieved a high specificity and sensitivity, detecting α-synuclein oligomers with a sub-peak current ratio method and a detection limit of 2.2 pM, distinguishing patient cohorts from healthy controls.

Nanopore-based single-molecule detection, especially through techniques like RPS and RT-FAST, was proved to be highly effective in studying amyloid and α-synuclein aggregation pathways. These methods provide valuable insights into the structural and kinetic properties of misfolded proteins, helping to distinguish between monomers, oligomers, and fibrils based on their unique translocation signals. The sensitivity and adaptability of nanopores, combined with innovations, like the RT-FAST assay, enable precise detection, even at low concentrations, making them indispensable for early diagnosis and for advancing our understanding of the molecular mechanisms driving neurodegenerative diseases. These innovations underline the potential of nanopipette platforms for clinical diagnostics and the molecular-level analysis of aggregation phenomena.

The development of nanopore-based techniques represents a significant advancement in the study of amyloid and α-synuclein aggregation pathways. Developed methods allow for the real-time, label-free, and ultrasensitive detection of protein aggregates, offering insights into the structural and kinetic properties of monomers, oligomers, and fibrils. As these technologies continue to evolve, they hold great promise for understanding the molecular mechanisms of neurodegenerative diseases and facilitating early diagnosis.

## 5. Conclusions, Challenges, and Potentials of Nanopipette-Based Nanopore Technology

In this review, we discuss the theoretical principles of the nanopipette-based nanopore detection method, important parameters of the method, and its application as a research tool for sensing biomolecules using selective carriers, including DNA and NPs, for β-amyloid and α-synuclein characterization. A nanopipette in RPS as a research tool has advantages with low-cost and easy fabrication, comparative durability, surface functionalization for different purposes, and high compatibility with other technologies that provide a wide range of applications.

This field still has challenges that need to be overcome, such as the rapid translocation of biomolecules, its folding or bumping, and complexity of protein–surface interactions. However, continued scientific and commercial interest and a growing number of studies make it possible to improve this ultrasensitive and universal analytical method for biomolecules detection.

One of the primary challenges is to enhance the specificity of nanopore signals for analyzing complex biological samples. Currently, the current is often used as the main parameter for molecule identification, but it lacks sufficient uniqueness for differentiating various molecule types, especially in complex biological samples.

Traditional molecule capture in nanopores relies on electrophoretic forces, which limits the simultaneous detection of molecules with different charges. The use of EOF enables the capture of neutral or oppositely charged molecules, but the charge density inside nanopores can restrict the range of detectable compounds. Developing charge-independent capture mechanisms is essential to broaden the spectrum of identifiable molecules, including proteins and polysaccharides. For analyzing molecules with a short lifetime, it is critical to improve the capture efficiency and temporal resolution.

The real-time detection of biomolecules in single cells using nanopipette-based nanopores is an emerging frontier in single-cell analysis. However, it presents several significant challenges. The dynamic nature of cellular environments poses one of the greatest challenges, as biomolecular concentrations fluctuate rapidly due to ongoing metabolic processes, signaling events, and external stimuli. This constant variability makes it difficult to capture and interpret transient molecular events with the precision required for real-time analysis.

Another challenge lies in the inherently low concentrations of many intracellular biomolecules, such as neurotransmitters and metabolites, which complicates their detection. Current nanopipette technology must overcome limitations in sensitivity to detect these molecules reliably. The intracellular environment further adds complexity, as non-specific interactions between molecules and the nanopore surface can influence accurate measurements. The complexity of nanopipette functionalization can limit widespread adoption, while nonspecific adsorption and variability in experimental conditions can impact reproducibility. Additionally, extending these methods to study heterogeneous and multi-component systems remains a critical goal. Addressing these limitations will require the further integration of computational modeling, advanced materials, and scalable fabrication techniques. While nanopipette fabrication is relatively inexpensive, scaling up production for widespread clinical use poses economic and logistical challenges.

Looking forward, nanopipette-based platforms have the potential to revolutionize the field of molecular diagnostics by enabling portable, cost-effective, and highly sensitive devices for detecting biomarkers. The unique combination of high sensitivity, spatial resolution, and multifunctionality positions it as a promising technology with numerous potential applications across biomedical research, diagnostics, and therapeutics.

In the field of diagnostics, nanopipette-based nanopores hold great promise for point-of-care applications. Their compact size and compatibility with portable systems make them ideal for developing affordable, user-friendly diagnostic tools. This potential is especially relevant for detecting biomarkers in low concentrations, such as circulating tumor DNA, specific RNA species, β-amyloid, and α-synuclein. Integration with microfluidics and automation could further expand the accessibility and scalability of nanopipette-based diagnostic platforms. Expanding their applications to other aggregation-prone proteins, leveraging multiplexing capabilities, and improving the compatibility with biological matrices will further enhance their utility.

The integration of nanopipette technology with artificial intelligence offers another important direction. Advanced algorithms could enhance signal interpretation, identify patterns in complex data, and provide predictive insights for both research and clinical applications. This combination of high-resolution sensing with data-driven analytics has the potential to uncover new biological mechanisms and optimize diagnostic workflows.

The development of multiplexed nanopipette arrays and their incorporation into high-throughput systems could enable the simultaneous analysis of multiple analytes, such as different types of biomolecules or cells. This approach would not only increase the efficiency but also broaden the scope of applications from large-scale population studies to personal medicine.

## Figures and Tables

**Table 1:** Recent advances in bare nanopipette-based nanopore detection over the past 5 years.

Material	Diameter	Analyte	Principles	Ref.
Borosilicate glass	5, 48 nm	λ-DNA	Development of theory and experiments underpinning the translocation mechanism, pulse direction and shape through the fine control of chemical, physical, and electronic parameters.	[89]
Quartz glass	~160 nm	DNA-origami	Single-molecule detection of supramolecular DNA assemblies using nanopipette, revealing ion current perturbations that fingerprint different assembly states, from monomer building blocks to multimers.	[90]
Borosilicate glass	22–46 nm	λ-DNA	This study investigates high-frequency capacitive noise in borosilicate nanopores, analyzing surface area effects by varying the submersion depth. IRMS values show depth-dependent noise performance, SNR, and λ-DNA capture frequency near the liquid–air interface.	[91]
Quartz glass	40–80 nm	Supercoiled plasmid DNA	Analysis of ECD quantifies branch sizes and positions on supercoiled plasmid DNA. Quartz nanopores demonstrated the capability to resolve multiple plasmid conformations (linear, circular, and supercoiled). Enzyme-dependent linearization of supercoiled DNA was determined achieving strong correlation with gel electrophoresis data.	[101]
Quartz glass	8 nm	Cytochrome c, protein G, trypsin, proteinase K, hemoglobin, bovine serum albumin, catalase, ferritin, α-macroglobulin, and other	Enhancing the SNR in nanopipette-based protein sensing to enable high-bandwidth molecular weight profiling.	[102]
Quartz glass	34, 67 nm	GOD	Detection of GOD by nanopipette.	[92]
Quartz glass	18.4 nm	GOD	Precise control of single GOD molecule movement inside a nanopipette.	[93]
Quartz glass	30–50 nm	GAD65 antigen and GADAb antibody	Detection of GAD65 antigen and antibody GADAb for the diagnosis and prognosis of type 1 diabetes.	[94]
Quartz glass	25 nm	Action dynamics	Monitoring of polymerization of actin in real-time by nanopipette.	[95]
Quartz glass	8–50 nm	Cas9 and Cas9d10a	Detection of complex Cas9d10a + sgRNA.	[103]

**Table 2:** Recent advances in nanopipette-based nanopore detection using a selective carrier or molecular probe over the past 5 years.

Analyte	Diameter of Nanopore in Quartz Nanopipette	Carrier/Molecular Construction	Limit of Detection	Principles	Ref.
AChE	30 nm	TDN–aptamer complex	-	- Highly sensitive detection of AChE using TDN. - Detection of AChE in serum.	[110]
ATP	50 nm	TDN–aptamer complex	-	- TDN enhances nanopipette detection sensitivity and stability. - TDN enables ATP sensing, undetectable without amplification. - TDN expands large-nanopore applications for biomolecule analysis.	[111]
Lysozyme	21 ± 4 nm	AuNPs modified with LBA	-	- Using AuNP-LBA for selective protein detection. AuNP-LBA bound to lysozyme produces characteristic two-step ion current signals that can distinguish between free AuNPs, AuNP-LBA, and AuNP-LBA-lysozyme complexes.	[112]
mRNA-141	~25 nm	AuNPs modified with aptamer	10^−12^ M	- High specificity in detecting miR-141-3p, a prostate cancer biomarker, using NP dimerization triggered by miRNA presence.	[113]
Procalcitonin	~25 nm	AuNPs modified with antibody	0.12 ng/mL	- Wide range of biomarker targets by functionalizing NPs with antibodies or aptamers.	[113]
CEA	~31 nm	Fe_3_O_4_-AuNPs functionalized with CEA aptamer	0.6 ng/mL	- Apt-MNPs are used to capture CEA from the sample. After magnetic separation, CEA-Apt-MNPs complexes are detected in the nanopore, creating deeper blocking current signals.	[114]
Dopamine, serotonin, and K^+^	25 nm	AuNPs (10, 20 nm) functionalized with PEG-SH or aptamers	12.5 nM (dopamine), 100 pM (serotonin), 900 nM (K^+^ ions)	- The unique double-barrel construction confines the analyte translocation path, reducing the speed and increasing detection precision.	[115]
Thrombin	21 nm	DNA carrier (48.5 kbp), modified with MB	0.5 nm	- Combination of electrical and optical detection for the label-free identification of small biomolecules, enabling synchronized single-molecule assays. - MCs increase the detection time within the nanopore, enhancing the SNR and detection accuracy for small biomolecules. - Reliable detection in challenging biological fluids (serum and urine).	[116]
miRNA: let-7a, miR-375, and miR-141	22 ± 3 nm	DNA carrier (5.6, 10, 38.5 kbp), modified with MB	~10 fM (miR-375 miR-141)	- Multiplexed detection of miRNAs using an electro-optical approach. - DNA carriers with MBs are used for the simultaneous detection of several miRNAs.	[37]
SARS-CoV-2 proteins (S and N proteins) and RNA fragments from ORF1b, S, and N genes	12 nm	DNA MC (10 kbp for proteins, 9.1 kbp for RNA) functionalized with aptamers or complementary ssDNA sequences	0.2 pM (S and N proteins) 0.01 RNA copies/µL	- Simultaneous detection of SARS-CoV-2 proteins and RNA fragments in unprocessed clinical samples, including saliva and nasal swabs. - High specificity achieved by using encoded DNA probes that generate distinct ion current signatures for bound analytes.	[41]
A29 protein of mpox virus (MPXV)	10 nm	Aptamer-grafted DNA carrier (9.1 kbp)	11 fM	- Detection of MPXV A29 protein at 11 fM with a range of 100 fM to 10 nM, suitable for rare protein detection in fluids. - Accurately distinguishes MPXV A29 from similar viral proteins, minimizing false positives. - Works effectively in serum and saliva, with strong A29 detection and minimal non-specific binding.	[46]
eGFP SfCherry, mIFP, and antigen–antibody complexes	12 nm	SUPs genetically fused to target proteins	74 pM	- SUPs slow down protein translocation through nanopores by up to 44.7-fold, enabling the detailed analysis of ionic current subpeaks. - The platform achieves a significant increase in protein capture rates, up to 136-fold compared with uncharged variants.	[44]
DNA fragments encoding the L1 gene of HPV18, a biomarker for cervical cancer	20 nm	TDN, triggered by CRISPR-Cas12a trans-cleavage activity	3 nM and linear range of 0.5–10 nM	- Combines CRISPR-Cas12a trans-cleavage activity with TDN for sensitive and indirect detection of small DNA fragments. - High selectivity, showing 5:1 discrimination between HPV18 and non-target DNA (e.g., HPV16, HIV).	[119]

## Abbreviations

4-MPBA	4-mercaptophenylboronic acid
AChE	Acetylcholinesterase
AD	Alzheimer’s disease
Aβ_42_	β-amyloid
BSA	Bovine serum albumin
CEA	Carcinoembryonic antigen
ECD	Excess charge deficit
ELISA	Enzyme-linked immunosorbent assay
EOF	Electroosmotic force
EPF	Electrophoretic force
GADAb	Glutamate decarboxylase
GOD	Glucose oxidase
ICR	Ionic current rectification
IgG	Immunoglobulin G
IRMS	Current root mean square
MB	Molecular beacon
MNPs	Magnetic nanoparticles
NexFT	Nanopore extended field-effect transistor
NPs	Nanoparticles
PPy	Polypyrrole
qRT-PCR	Quantitative reverse transcription polymerase chain reaction
RT-FAST	Real-time fast amyloid seeding and translocation
RPS	Resistive pulse sensing
SECM	Scanning electrochemical microscopy
SICM	Scanning ion-conductance microscopy
SNR	Signal-to-noise ratio
SUP	Supercharged unstructured polypeptides
TDN	Tetrahedral DNA nanostructure
